# Author Correction: Cryo-EM structures of ryanodine receptors and diamide insecticides reveal the mechanisms of selectivity and resistance

**DOI:** 10.1038/s41467-024-53939-2

**Published:** 2024-11-13

**Authors:** Lianyun Lin, Changshi Wang, Wenlan Wang, Heng Jiang, Takashi Murayama, Takuya Kobayashi, Hadiatullah Hadiatullah, Yu Seby Chen, Shunfan Wu, Yiwen Wang, Henryk Korza, Yucheng Gu, Yan Zhang, Jiamu Du, Filip Van Petegem, Zhiguang Yuchi

**Affiliations:** 1 Tianjin Key Laboratory for Modern Drug Delivery and High-Efficiency, Frontiers Science Center for Synthetic Biology, School of Pharmaceutical Science and Technology, Faculty of Medicine, Tianjin University, Tianjin, China; 2 Haihe Laboratory of Sustainable Chemical Transformations, Tianjin, China; 3 Institute of Plant and Food Science, Department of Biology, Southern University of Science and Technology, Shenzhen, Guangdong China; 4 Department of Cellular and Molecular Pharmacology, Juntendo University Graduate School of Medicine, Tokyo, Japan; 5 Department of Biochemistry and Molecular Biology, Life Sciences Institute, University of British Columbia, Vancouver, BC Canada; 6 College of Plant Protection, State & Local Joint Engineering Research Center of Green Pesticide Invention and Application, Nanjing Agricultural University, Nanjing, Jiangsu China; 7 Syngenta Jealott’s Hill International Research Centre, Bracknell, Berkshire UK; 8 Guangdong Laboratory for Lingnan Modern Agriculture (Shenzhen Branch), Agricultural Genomics Institute at Shenzhen, Chinese Academy of Agricultural Sciences, Shenzhen, Guangdong China

**Keywords:** Cryoelectron microscopy, Calcium channels

**Correction to:** *Nature Communications* (2024)10.1038/s41467-024-53490-0,published online 20 October 2024

In this article the affiliation details for Changshi Wang were incorrectly given as ‘Department of Biochemistry and Molecular Biology, Life Sciences Institute, University of British Columbia, Vancouver, British Columbia, Canada’ but should have been ‘Institute of Plant and Food Science, Department of Biology, Southern University of Science and Technology, Shenzhen, Guangdong, China’. The original article has been corrected.

