# Supplementary material for: Cryo-EM structures of ryanodine receptors and diamide insecticides reveal the mechanisms of selectivity and resistance
Source: Nat Commun. 2024 Oct 20;15:9056. doi: 10.1038/s41467-024-53490-0 (PMC11491487; doi:10.1038/s41467-024-53490-0)

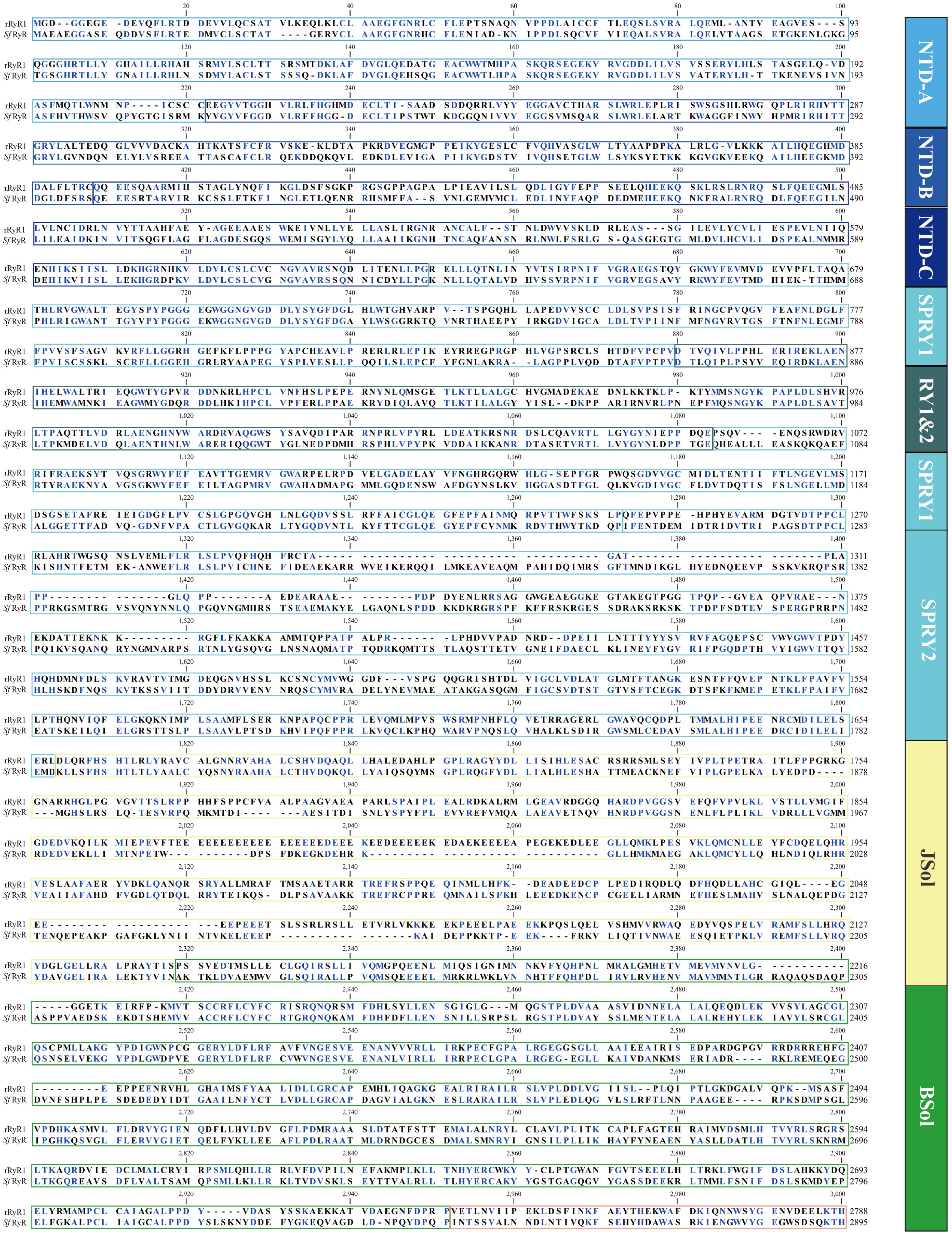
rRyR1
SfRyR
NTD-A 59.7%
NTD-B 47.1%
NTD-C 51.5%
SPRY1 52.2%
RY1&2 53.4%
SPRY1 46.6%
SPRY2 29.0%
JSol 35.7%
BSol 48.5%

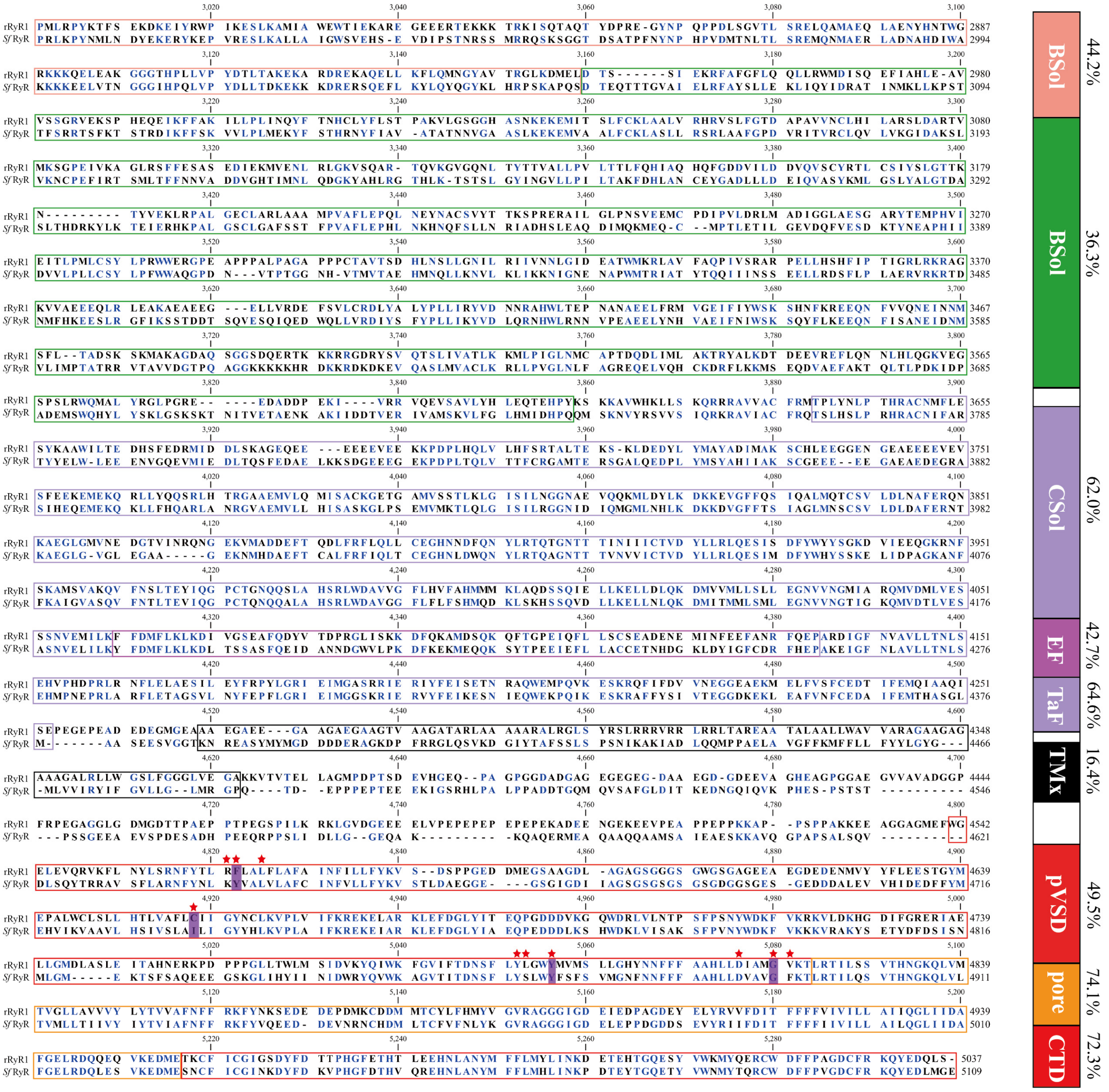

Supplementary Figure 1. Sequence alignment between rRyR1 and *Sf* RyR. Different domains are outlined in boxes of various colors, and the sequence identities of these domains are displayed on the right side. Amino acids that interact with diamides are marked with asterisks, while the resistance mutation sites are highlighted in purple.

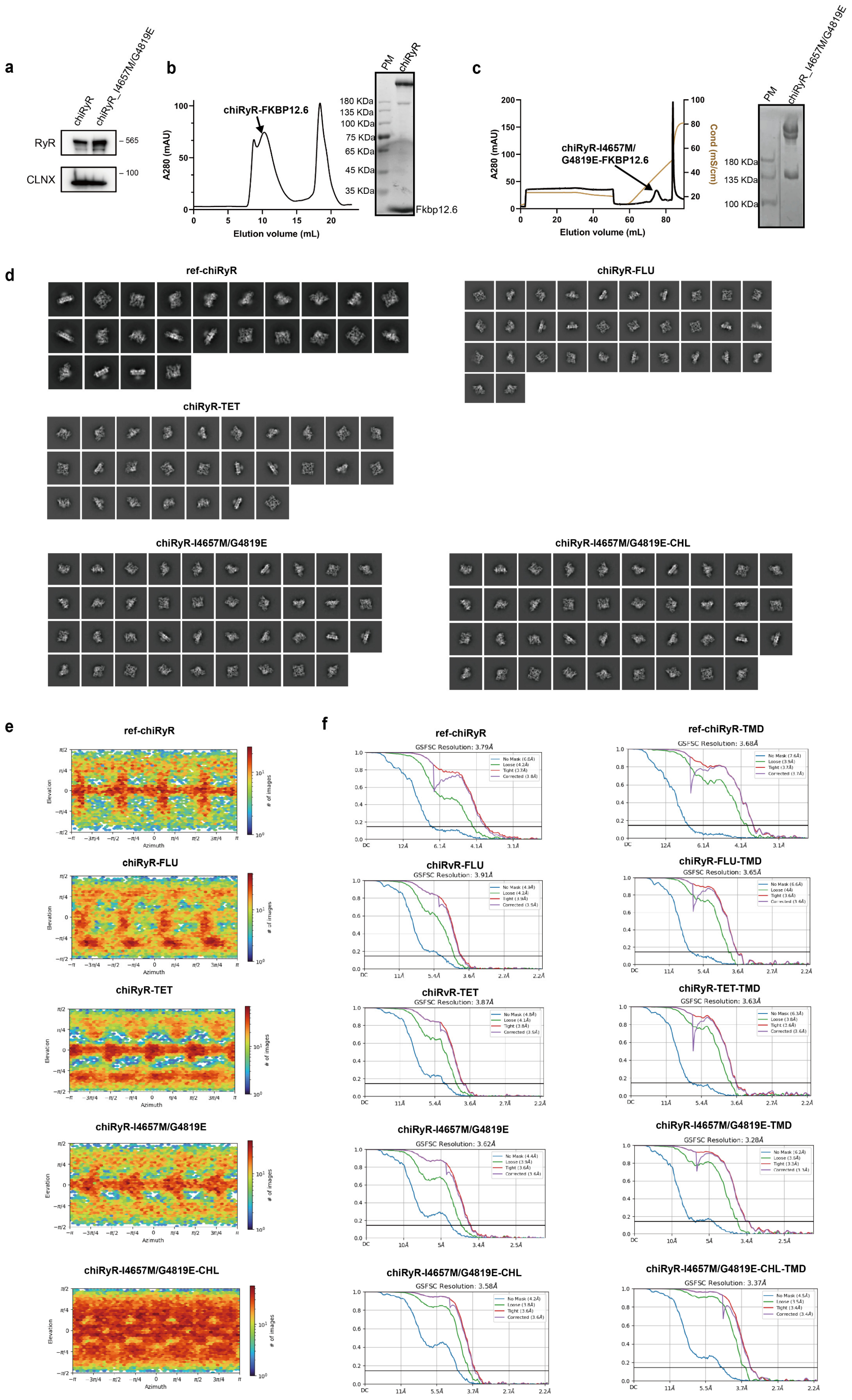
a
chiRyR
chiRyR_I4657M/G4819E
RyR
CLNX
565
100
b
chiRyR-FKBP12.6
A280 (mAU)
Elution volume (mL)
PM
chiRyR
180 KDa
135 KDa
100 KDa
75 KDa
65 KDa
45 KDa
35 KDa
Fkbp12.6
c
chiRyR-I4657M/
G4819E-FKBP12.6
A280 (mAU)
Cond (mS/cm)
Elution volume (mL)
PM
chiRyR_I4657M/G4819E
180 KDa
135 KDa
100 KDa
d
ref-chiRyR
chiRyR-FLU
chiRyR-TET
chiRyR-I4657M/G4819E
chiRyR-I4657M/G4819E-CHL
e
ref-chiRyR
chiRyR-FLU
chiRyR-TET
chiRyR-I4657M/G4819E
chiRyR-I4657M/G4819E-CHL
Elevation
Azimuth
# of images
f
ref-chiRyR
GSFSC Resolution: 3.79Å
ref-chiRyR-TMD
GSFSC Resolution: 3.68Å
chiRyR-FLU
GSFSC Resolution: 3.91Å
chiRyR-FLU-TMD
GSFSC Resolution: 3.65Å
chiRyR-TET
GSFSC Resolution: 3.87Å
chiRyR-TET-TMD
GSFSC Resolution: 3.63Å
chiRyR-I4657M/G4819E
GSFSC Resolution: 3.62Å
chiRyR-I4657M/G4819E-TMD
GSFSC Resolution: 3.28Å
chiRyR-I4657M/G4819E-CHL
GSFSC Resolution: 3.58Å
chiRyR-I4657M/G4819E-CHL-TMD
GSFSC Resolution: 3.37Å

Supplementary Figure 2. Purification and cryo-EM analysis of chiRyRs. a Western blot showing the expression of chiRyR and chiRyR-I4657M/G4819E in HEK293 cells. CLNX protein was used as the internal control. Source data are provided as a Source Data file. **b** Purification of chiRyR. The elution profile of chiRyR by gel-filtration chromatography using a Superose 6 Increase column is shown in the left panel, and SDS-PAGE showing purified chiRyR is in the right panel. Source data are provided as a Source Data file. **c** Purification of chiRyR- I4657M/G4819E. The elution profile of chiRyR-I4657M/G4819E by anion exchange chromatography using a HiRes Q 5/50 column is shown in the left panel, and SDS-PAGE showing purified chiRyR-I4657M/G4819E is in the right panel. **d** Representative two-dimensional class averages for ref-chiRyR, chiRyR-FLU, chiRyR-TET, chiRyR-I4657M/G4819E, and chiRyR-I4657M/G4819E-CHL. **e** The orientation distributions of the particles used for the final reconstructions. **f** Summary of the Fourier shell correlation (FSC) curves generated by CryoSPARC for global refinement (left) and TMD local refinement (right).

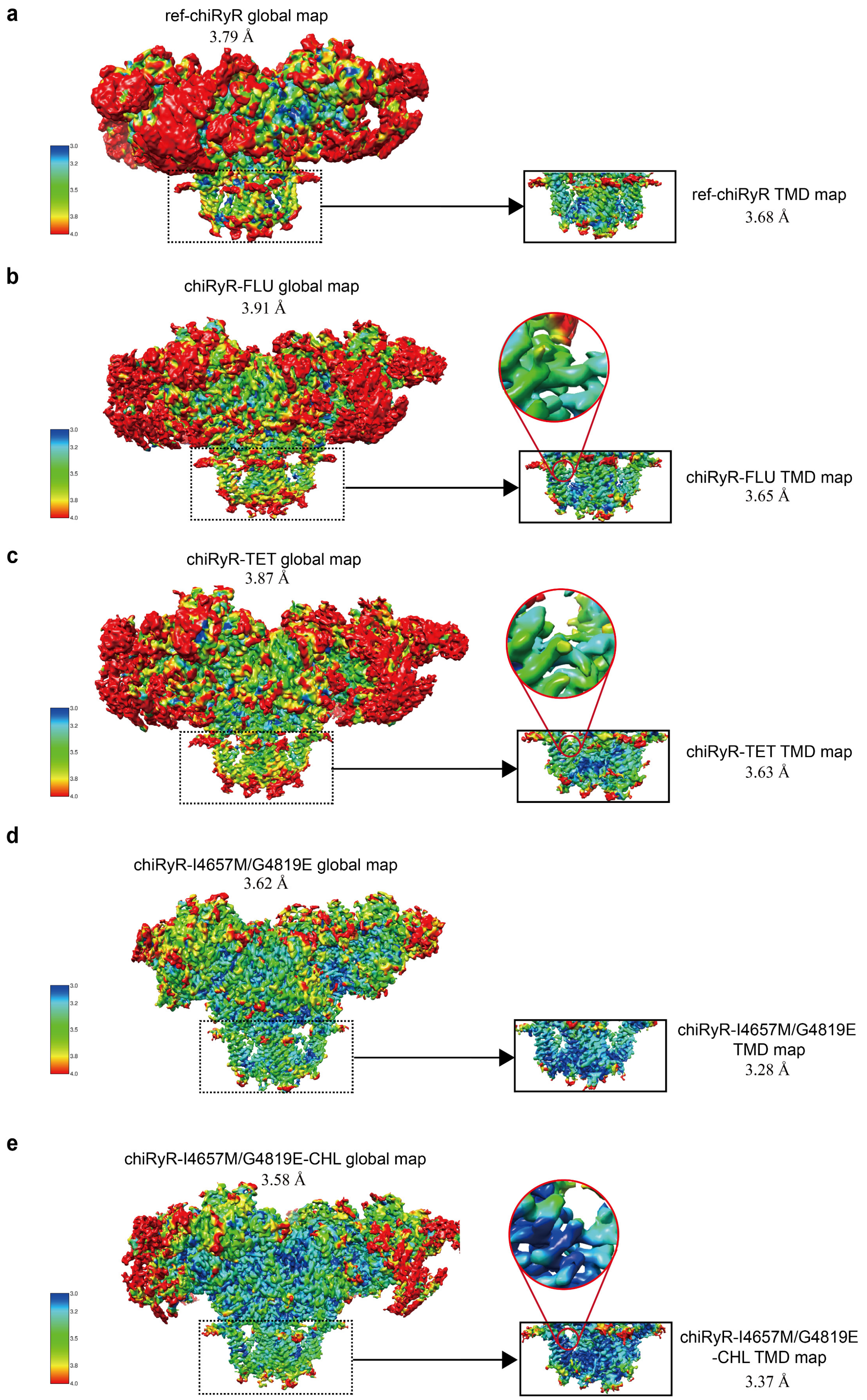
a
ref-chiRyR global map
3.79 Å
ref-chiRyR TMD map
3.68 Å
b
chiRyR-FLU global map
3.91 Å
chiRyR-FLU TMD map
3.65 Å
c
chiRyR-TET global map
3.87 Å
chiRyR-TET TMD map
3.63 Å
d
chiRyR-I4657M/G4819E global map
3.62 Å
chiRyR-I4657M/G4819E
TMD map
3.28 Å
e
chiRyR-I4657M/G4819E-CHL global map
3.58 Å
chiRyR-I4657M/G4819E
-CHL TMD map
3.37 Å

Supplementary Figure 3. Local resolution estimates. Local resolution estimation is shown by colored global and locally refined maps for ref-chiRyR (a), chiRyR-FLU (b), chiRyR-TET (c), chiRyR-I4657M/G4819E (d), and chiRyR-I4657M/G4819E-CHL (e). The color bar indicates resolution in angstroms (Å). The ligands in chiRyR-FLU, chiRyR-TET, and chiRyR-I4657M/G4819E-CHL are shown in zoomed-in insets.

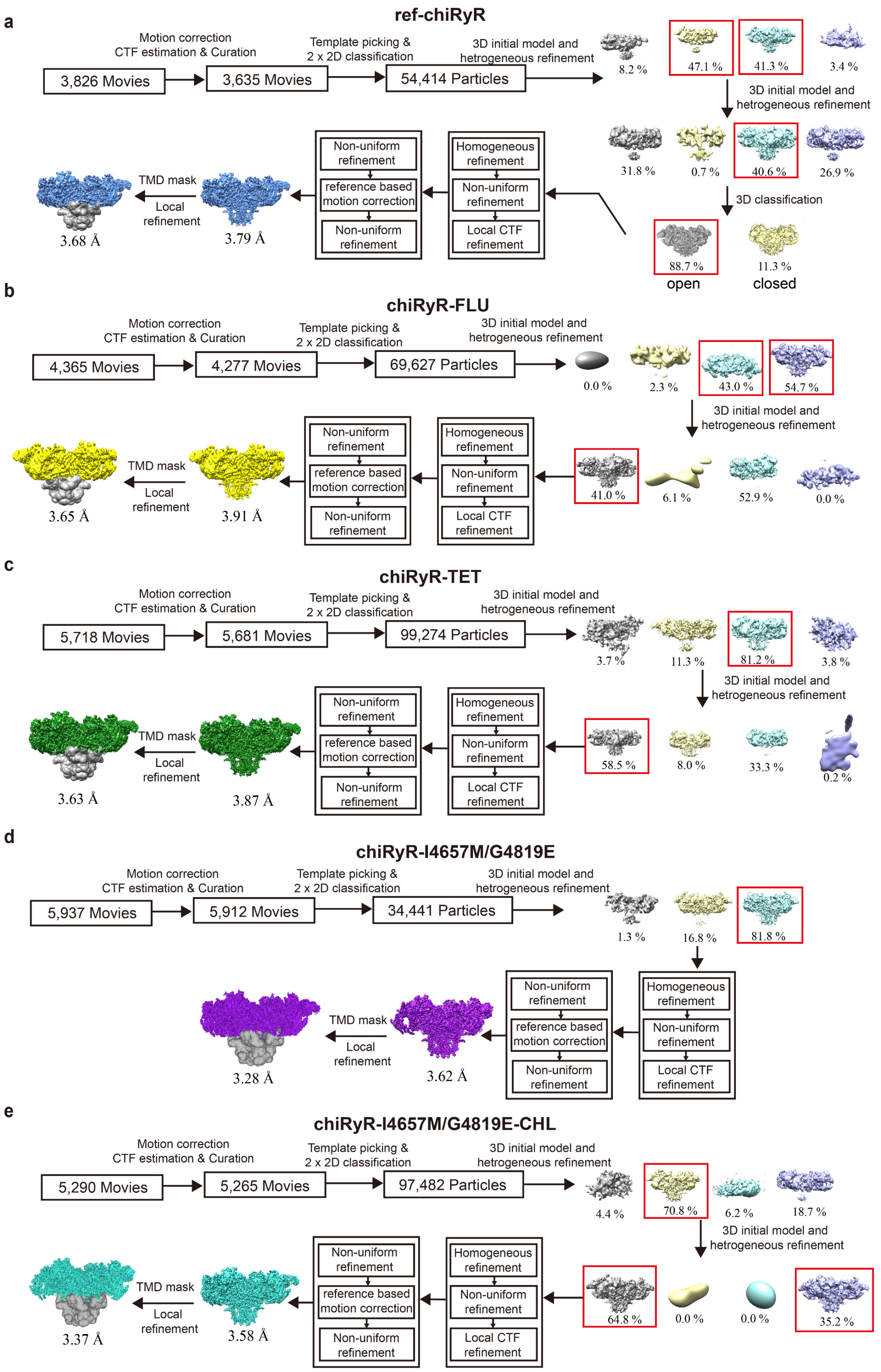

Supplementary Figure 4. Cryo-EM processing pipeline. Cryo-EM data collection, 3D classification, and refinement schemes for ref-chiRyR (a), chiRyR-FLU (b), chiRyR-TET (c), chiRyR-I4657M/G4819E (d), and chiRyR-I4657M/G4819E-CHL (e).

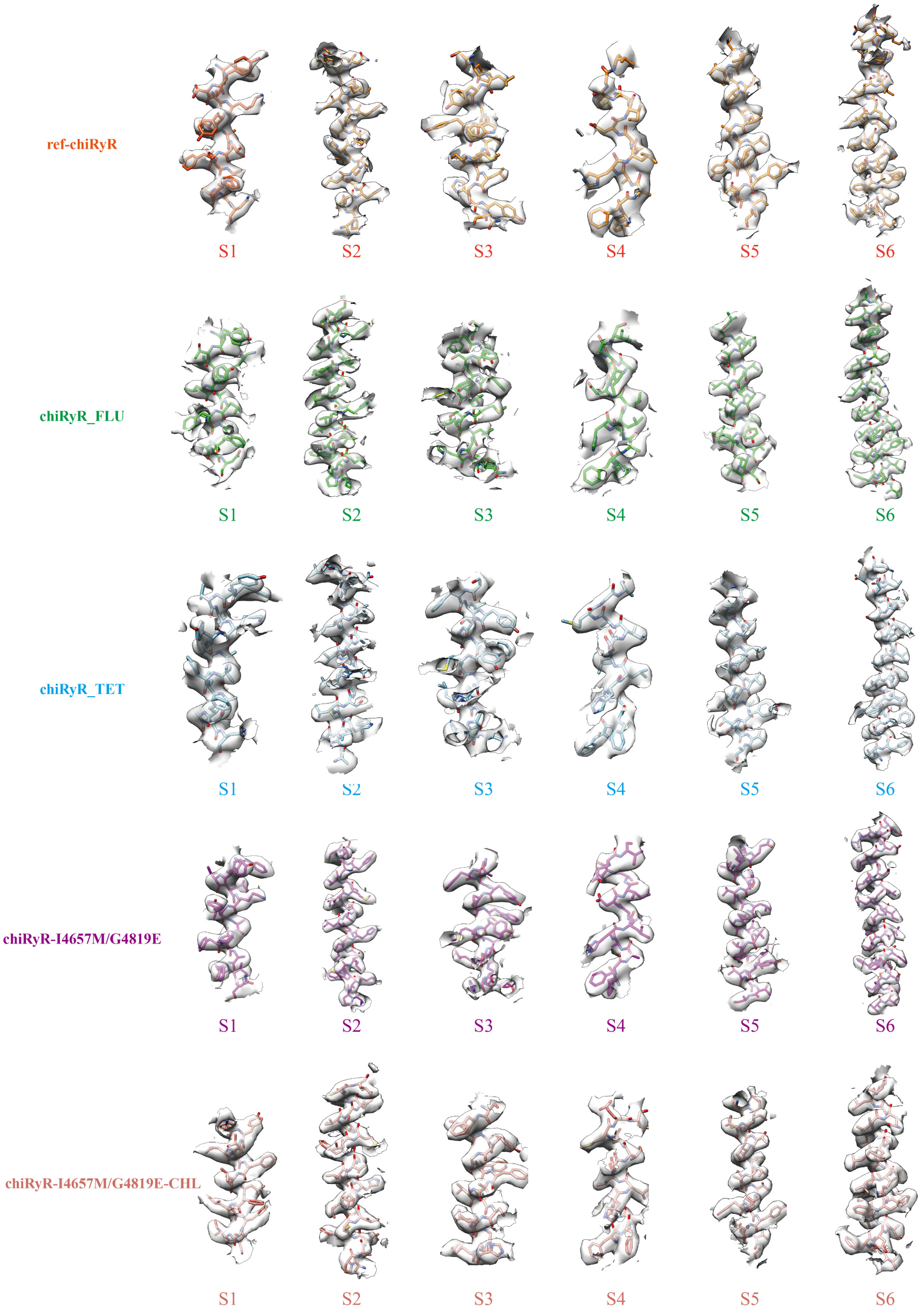

Supplementary Figure 5. The density of the S1-S6 helices from different chiRyRs.

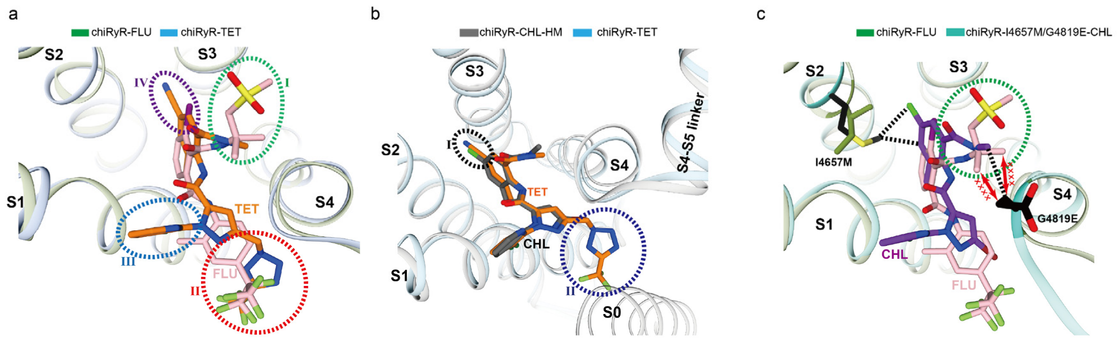

Supplementary Figure 6. Comparison of the diamide binding poses. a The comparison of the diamide binding poses between chiRyR-FLU and chiRyR-TET reveals four major differences. **b** The comparison of the diamide binding poses between chiRyR-CHL (chiRyR-CH-HM: a homology model based on rRyR1-CHL (PDB ID: 6M2W)) and chiRyR-TET reveals two major differences. **c** The comparison of the diamide binding poses between chiRyR-FLU and chiRyR-I4657M/G4819E-CHL shows that FLU would have clash with the Glu4819.

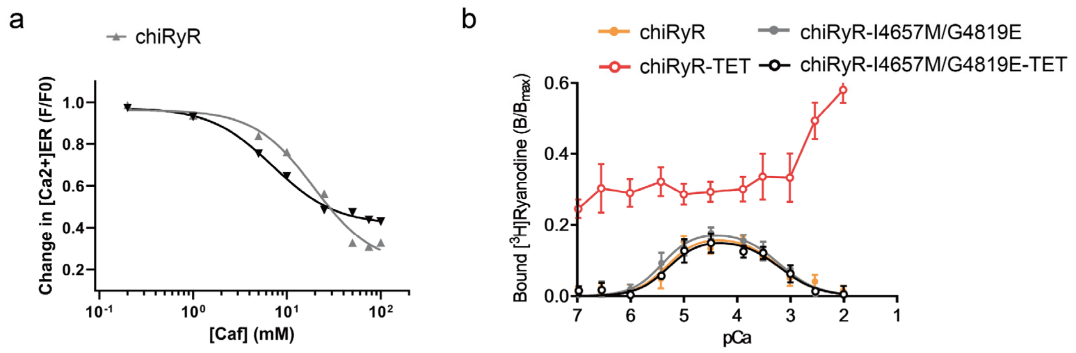

Supplementary Figure 7. Functional characterization of chiRyR and chiRyR-I4657M/G4819E. a The dose-dependent curves of caffeine show that the resistance mutations does not significantly change the caffeine sensitivity of chiRyR. The EC_{50} values for chiRyR and chiRyR-I4657M/G4819E are 19.5 mM and 7.1 mM, respectively (Supplementary Table 4). Source data are provided as a Source Data file. **b** Ca^{2+}-dependent [^{3}H]ryanodine binding of chiRyR and chiRyR-I4657M/G4819E in the absence or presence of TET. The biphasic Ca^{2+}-dependency of chiRyR-I4657M/G4819E shows no significant difference compared to that of chiRyR, with EC_{50} values for Ca^{2+} 5.0 ± 1.1μM (chiRyR) and 4.4 ± 2.4 μM (chiRyR-I4657M/G4819E) and IC_{50} values for Ca^{2+} are 0.67 ± 0.37mM (chiRyR) and 0.59 ± 0.19 mM (chiRyR-I4657M/G4819E) (mean ± SD, n=4). 1 μM TET can promote [^{3}H]ryanodine binding in chiRyR but not in chiRyR-I4657M/G4819E. Source data are provided as a Source Data file.

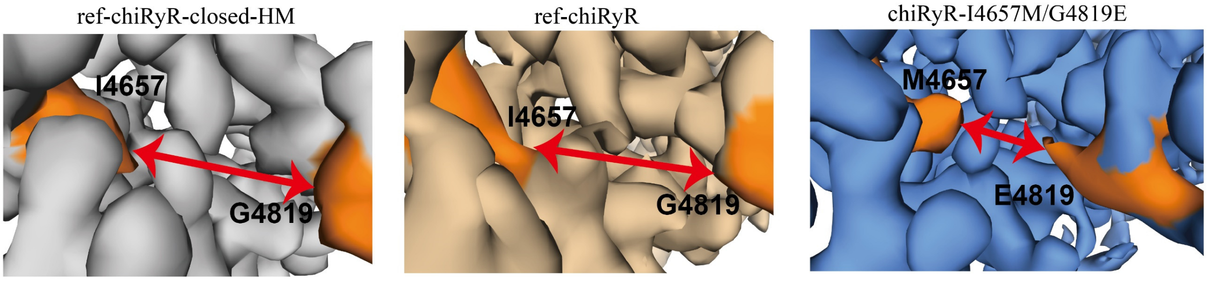

Supplementary Figure 8. Comparison of the diamide binding pockets between wild-type and resistant RyRs. Comparison of the density maps of the diamide-binding pocket between the closed-state ref-chiRyR (ref-chiRyR-closed-HM: a homology model based on closed rRyR1(PDB ID: 5TAQ), ref-chiRyR and chiRyR-I4657M/G4819E-CHL. The density mao of ref-chiRyR-closed-HM was generated by the molmap module in UCSF Chimera. The density maps are shown at the 5σ level. The two resistance mutation sites are colored in orange, and the distances between them are indicated by red arrows.

Supplementary Table 1. EC_{50} values of diamide insecticides measured by time-lapse fluorescence assay using stable cell lines expressing RyRs from different species

Diamide	RyR variants	EC_{50}[a]	95% CI	R^2
FLU	rRyR1	4.5 µM	2.2 µM– 14.0 µM	0.874
	Sf RyR	59.3 nM	45.6 nM – 76.2 nM	0.984
	chiRyR	25.1 nM	20.7 nM – 30.6 nM	0.996
TET	rRyR1	7.6 µM	5.8 µM – 10.0 µM	0.978
	Sf RyR	174.5 nM	130.2 nM - 224.7 nM	0.979
	chiRyR	46.1 nM	35.4 nM – 62.8 nM	0.935

a: n = 3.

Supplementary Table 2. EC_{50} values of diamide insecticides measured by time-lapse fluorescence assay using stable cell lines expressing different rRyR1 mutants

Diamide	RyR variants	EC_{50}[a]	95% CI	R^2
FLU	rRyR1	4.5 µM	2.2 µM– 14.0 µM	0.874
	rRyR1 R4563K	3.9 µM	3.5 µM – 4.3 µM	0.995
	rRyR1 F4564Y	26.1 µM	21.6 µM – 33.4 µM	0.985
	rRyR1 C4657I	8.9 µM	8.2 µM – 9.6 µM	0.995
	rRyR1 L4792S	208.9 nM	172.7 nM – 250.4 nM	0.976
CHL	rRyR1	4.9 µM	4.5 µM – 5.4 µM	0.996
	rRyR1 R4563K	62.6 nM	52.8 nM – 74.0 nM	0.985
	rRyR1 F4564Y	555.7 nM	478.9 nM – 645.0 nM	0.993
	rRyR1 C4657I	167.3 nM	153.2 nM – 183.0 nM	0.994
	rRyR1 L4792S	44.8 nM	42.7 nM – 47.2 nM	0.994

a: n = 3.

Supplementary Table 3. Cryo-EM data collection, refinement and validation statistics

Protein	chiRyR			chiRyR-I4657M/G4819E	
Condition	5 mM caffeine<br>2 mM ATP<br>100 µM CaM1234<br><br>2% DMSO	5 mM caffeine<br>2 mM ATP<br>100 µM CaM1234<br>50 µM FLU in 2% DMSO	5 mM caffeine<br>2 mM ATP<br>100 µM CaM1234<br>50 µM TET in 2% DMSO	5 mM caffeine<br>2 mM ATP<br>100 µM CaM1234<br><br>2% DMSO	5 mM caffeine<br>2 mM ATP<br>100 µM CaM1234<br>625 µM CHL in 5% DMSO
PDB ID	8XLF	8XJI	8XKH	8XLH	8Y40
EMDB ID	EMD-38447, EMD-60900 for TMD	EMD-38398, EMD-38551 for TMD	EMD-38417, EMD-38553 for TMD	EMD-38448, EMD-60899 for TMD	EMD-38446, EMD- 60901 for TMD
**Data collection and processing**					
Microscope	FEI Titan Krios				
Detector	Gatan K3 direct electron detector				
Magnification	×22,500				
Volotage (kV)	300				
Electron exposure ($e^-/Å^2$)	50				
Defocus range (µm)	1.0-2.4				
Pixel size (Å)	1.06				
Initial particle images	54,414	69,627	99,274	34,441	97,482
Final particle images	19,505	27,906	47,206	28,189	69,016
Map resolution (Å)	3.79	3.91	3.87	3.62	3.58
FSC threshold	0.143				
**Model building and refinement**					
Model composition					
Protein atoms	119,048	119,880	119,330	124,576	123,204
Ligands	16	20	20	16	20
R.M.S. deviations					
Bond length (Å)	0.008	0.007	0.007	0.009	0
Bond angles (°)	0.974	0.904	0.882	1.045	1.169
**Validation**					
MolProbity score	2.22	2.02	2.37	2.58	2.41
Clashscore	6.03	9.28	10.21	11.15	11.26
Rotamer outliers (%)	0.62	0.98	1.13	1.28	1.96
Ramachandran plot					
Favored (%)	89.86	94.13	92.36	84.61	87.19
Allowed (%)	9.90	5.51	6.65	14.26	11.78
Outlier (%)	0.62	0.35	1.00	1.24	1.02

Supplementary Table 4. EC_{50} values of diamide insecticides and caffeine measured by time-lapse fluorescence assay using stable cell lines expressing wild-type or resistant RyRs

Diamide	RyR variants	EC_{50}[a]	95% CI	R^2
FLU[b]	chiRyR	25.1 nM	20.1 nM – 30.6 nM	0.996
	chiRyR-I4657M/G4819E	> 300 μM	-	-
	Sf RyR	59.3 nM	45.6 nM – 76.2 nM	0.984
	Sf RyR_I4734M/G4891E	> 300 μM	-	-
CHL	chiRyR	1.6 nM	1.2 nM – 2.1nM	0.965
	chiRyR-I4657M/G4819E	14.6 μM	10.1 μM – 21.2 μM	0.893
	Sf RyR	5.5 nM	5.3 nM – 5.7 nM	0.998
	Sf RyR_I4734M/G4891E	9.1 μM	7.1 μM – 11.5 μM	0.982
Caffeine	chiRyR	19.5 mM	14.3 mM – 35.7 mM	0.983
	chiRyR-I4657M/G4819E	7.1 mM	4.6 mM – 11.7 mM	0.970

a: n = 3; b: Due to the solubility limit, the highest tested concentration of FLU was 300 μM.

Supplementary Table 5. EC_{50} values of diamide insecticides measured by [^{3}H]Ryanodine binding assay

	**chiRyR**[a]	**chiRyR-I4657M/G4819E**[a]
**CHL**	1.7 ± 0.3 nM	14.7 ± 1.6 μM
**TET**	11.1 ± 0.9 nM	87.8 ± 6.4 μM
**FLU**	4.0 ± 1.4 nM	-

a: n = 4.

Supplementary Table 6. The LD_{50} values of two diamide insecticides in different Cas9 *Drosophila melanogaster*

Diamide	*Drosophila*	LD_{50}[a]	95% CI	R^2
FLU*	M4758I	10.4 μM	8.4 μM - 12.8 μM	0.973
	G4915E	AA.	-	-
CHL	M4758I	127.9 nM	107.8 nM – 152.8 nM	0.977
	G4915E	1.6 mM	460.1 μm – 30.2 mM	0.711

a: n = 30; AA.: All alive.

Supplementary Table 7. Primers for rRyR1 and *Sf* RyR mutations

Name	Sequence (5'→ 3')
rRyR1-R4563K-F	CTACACCCTGAAGTTCCTTGCC
rRyR1-R4563K-R	GGCAAGGAACTTCAGGGTGTAG
rRyR1-F4564Y-F	CACCCTGCGCTACCTTGCCCTCTTC
rRyR1-F4564Y-R	GAAGAGGGCAAGGTAGCGCAGGGTG
rRyR1-C4657I-F	GCCTTCCTCATCATCATCGG
rRyR1-C4657I-R	CCGATGATGATGAGGAAGGC
rRyR1-L4792S-F	GTTCCTGTACAGCGGCTGGTAC
rRyR1-L4792S-R	GTACCAGCCGCTGTACAGGAAC
rRyR1-I4657M-F	GTGGCCTTCCTCATGATCATCGGCTAC
rRyR1-I4657M-R	GTAGCCGATGATCATGAGGAAGGCCAC
rRyR1-G4819E-F	CATCGCCATGGAGGTCAAGACGCTG
rRyR1-G4819E-R	CAGCGTCTTGACCTCCATGGCGATG
*Sf*RyR-I4734M-F	GTGTCGCTCGCTATGCTCATCGGGT
*Sf*RyR-I4734M-R	ACCCGATGAGCATAGCGAGCGACAC
*Sf*RyR-G4891E-F	AGATGTCGCTGTCGAGTTCAAGACC
*Sf*RyR-G4891E-R	GGTCTTGAACTCGACAGCGACATCT

For Source Data

Supplementary Figure 2a uncropped blot

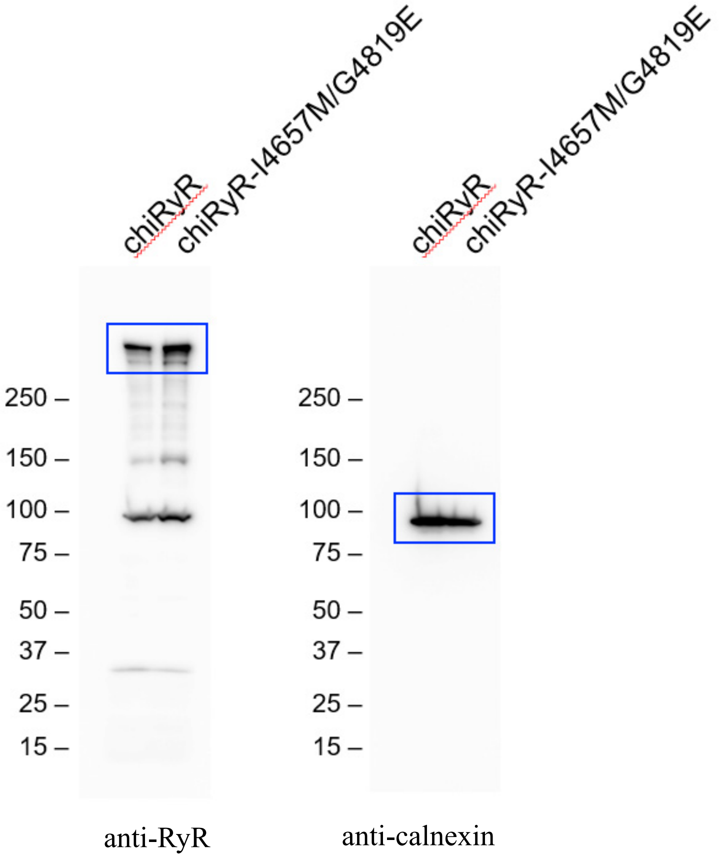

For Source Data

Supplementary Figure 2a uncropped gel

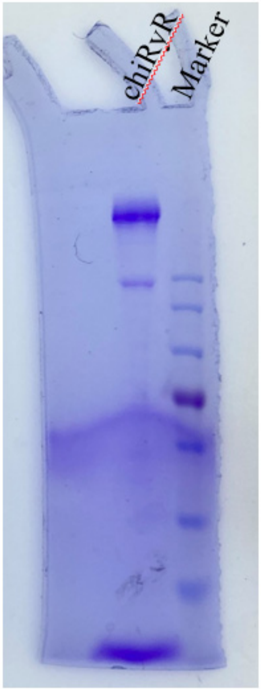

Supplementary Figure 2c uncropped gel

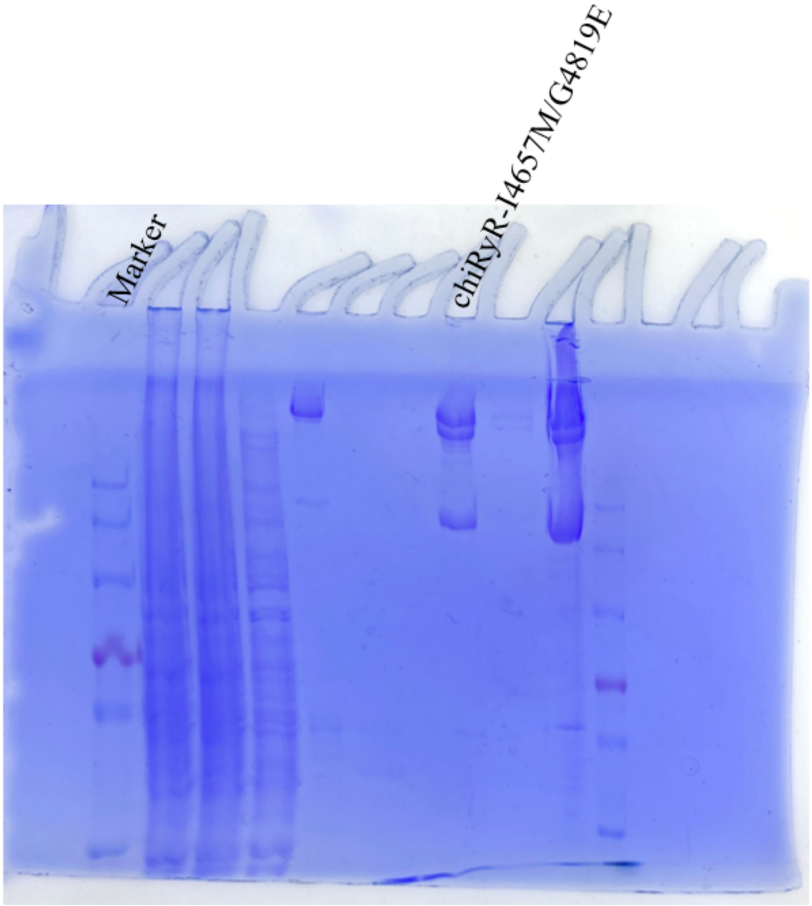

Supplement: Supplementary file 1 — Supplementary Information [file 41467_2024_53490_MOESM1_ESM.pdf]
