# Supplementary material for: Cryo-EM structures of ryanodine receptors and diamide insecticides reveal the mechanisms of selectivity and resistance
Source: Nat Commun. 2024 Oct 20;15:9056. doi: 10.1038/s41467-024-53490-0 (PMC11491487; doi:10.1038/s41467-024-53490-0)
Supplement: Supplementary file 2 — Reporting Summary [file 41467_2024_53490_MOESM2_ESM.pdf]

nature portfolio

Corresponding author(s): Filip Van Petegem, Zhiguang Yuchi.

Last updated by author(s): Aug 14, 2024

Reporting Summary

Nature Portfolio wishes to improve the reproducibility of the work that we publish. This form provides structure for consistency and transparency in reporting. For further information on Nature Portfolio policies, see our Editorial Policies and the Editorial Policy Checklist.

Statistics

For all statistical analyses, confirm that the following items are present in the figure legend, table legend, main text, or Methods section.

n/a	Confirmed	
☐	☒	The exact sample size (*n*) for each experimental group/condition, given as a discrete number and unit of measurement
☐	☒	A statement on whether measurements were taken from distinct samples or whether the same sample was measured repeatedly
☐	☒	The statistical test(s) used AND whether they are one- or two-sided<br>*Only common tests should be described solely by name; describe more complex techniques in the Methods section.*
☒	☐	A description of all covariates tested
☒	☐	A description of any assumptions or corrections, such as tests of normality and adjustment for multiple comparisons
☐	☒	A full description of the statistical parameters including central tendency (e.g. means) or other basic estimates (e.g. regression coefficient) AND variation (e.g. standard deviation) or associated estimates of uncertainty (e.g. confidence intervals)
☒	☐	For null hypothesis testing, the test statistic (e.g. *F*, *t*, *r*) with confidence intervals, effect sizes, degrees of freedom and *P* value noted<br>*Give P values as exact values whenever suitable.*
☒	☐	For Bayesian analysis, information on the choice of priors and Markov chain Monte Carlo settings
☒	☐	For hierarchical and complex designs, identification of the appropriate level for tests and full reporting of outcomes
☒	☐	Estimates of effect sizes (e.g. Cohen's *d*, Pearson's *r*), indicating how they were calculated

Our web collection on statistics for biologists contains articles on many of the points above.

Software and code

Policy information about availability of computer code

Data collection	Movie stacks for cryo-EM were collected using the automated collection system EPU (Thermo Fisher). FlexStation 3 fluorometer (Molecular Devices) data was collected using SoftMax Pro 7.0.3.
Data analysis	Cryosparc 4.4.1, Coot-0.8.9.2, PHENIX-1.16, Coot, UCSF Chimera 1.14, Graphpad Prism 8, Schrodinger 2017, and HOLE 2.0 were used to analyze the data in this study.

For manuscripts utilizing custom algorithms or software that are central to the research but not yet described in published literature, software must be made available to editors and reviewers. We strongly encourage code deposition in a community repository (e.g. GitHub). See the Nature Portfolio guidelines for submitting code & software for further information.

Data

Policy information about availability of data

All manuscripts must include a data availability statement. This statement should provide the following information, where applicable:

- Accession codes, unique identifiers, or web links for publicly available datasets
- A description of any restrictions on data availability
- For clinical datasets or third party data, please ensure that the statement adheres to our policy

The structures used in this paper have been previously published are deposited in the PDB database, including the pdb codes: 6M2W [http://doi.org/10.2210/pdb6M2W/pdb], 5TAL [http://doi.org/10.2210/pdb5TAL/pdb], and 5TAQ [http://doi.org/10.2210/pdb5TAQ/pdb].

Cryo-EM maps and structures generated in this study have been deposited in the EMDB and PDB databases, respectively, with the following accession codes:
ref-chiRyR: PDB 8XLF [http://doi.org/10.2210/pdb8XLF/pdb], EMDB EMD-38447 [https://www.ebi.ac.uk/pdbe/entry/emdb/EMD-38447] and EMD-60900 (TMD local, [https://www.ebi.ac.uk/pdbe/entry/emdb/EMD-60900]).
chiRyR-FLU: PDB 8XJI [http://doi.org/10.2210/pdb8XJI/pdb], EMDB EMD-38398 [https://www.ebi.ac.uk/pdbe/entry/emdb/EMD-38398] and EMD-38551 (TMD local, [https://www.ebi.ac.uk/pdbe/entry/emdb/EMD-38551])
chiRyR-TET: PDB 8XKH [http://doi.org/10.2210/pdb8XKH/pdb], EMDB EMD-38417 [https://www.ebi.ac.uk/pdbe/entry/emdb/EMD-38417] and EMD-38553 (TMD local, [https://www.ebi.ac.uk/pdbe/entry/emdb/EMD-38553])
chiRyR-I4657M/G4819E: PDB 8XLH [http://doi.org/10.2210/pdb8XLH/pdb], EMDB EMD-38448 [https://www.ebi.ac.uk/pdbe/entry/emdb/EMD-38448] and EMD-60899 (TMD local, [https://www.ebi.ac.uk/pdbe/entry/emdb/EMD-60899])
chiRyR-I4657M/G4819E-CHL: PDB 8Y40 [http://doi.org/10.2210/pdb8Y40/pdb], EMDB EMD-38908 [https://www.ebi.ac.uk/pdbe/entry/emdb/EMD-38908] and EMD-60901 (TMD local, [https://www.ebi.ac.uk/pdbe/entry/emdb/EMD-60901]) .
All the data needed to evaluate the conclusions are presented in the paper or the Supplementary Materials.

Research involving human participants, their data, or biological material

Policy information about studies with human participants or human data. See also policy information about sex, gender (identity/presentation), and sexual orientation and race, ethnicity and racism.

Reporting on sex and gender	This information has not been collected.
Reporting on race, ethnicity, or other socially relevant groupings	This information has not been collected.
Population characteristics	This information has not been collected.
Recruitment	This information has not been collected.
Ethics oversight	This information has not been collected.

Note that full information on the approval of the study protocol must also be provided in the manuscript.

Field-specific reporting

Please select the one below that is the best fit for your research. If you are not sure, read the appropriate sections before making your selection.

☒ Life sciences ☐ Behavioural & social sciences ☐ Ecological, evolutionary & environmental sciences

For a reference copy of the document with all sections, see nature.com/documents/nr-reporting-summary-flat.pdf

Life sciences study design

All studies must disclose on these points even when the disclosure is negative.

Sample size	No sample-size calculations were performed in this study. In the cell-based assay, several tens of cells are sufficient. Because [Ca2+]ER measurements is a biochemical assay with microsomes from many (>2x10^4) cells, three or more samples are sufficient. For Drosophila melanogaster assay, 30 flies are sufficient for each tested insecticide concentration.
Data exclusions	Data were not excluded from analysis.
Replication	Experimental findings were reliably reproduced. All experiments were successfully repeated three times on separate occasions.
Randomization	Randomization was not relevant to our study. Because there's no allocation of samples/organisms/participants involved in our study.
Blinding	Blinding was not relevant to our study.

Reporting for specific materials, systems and methods

We require information from authors about some types of materials, experimental systems and methods used in many studies. Here, indicate whether each material, system or method listed is relevant to your study. If you are not sure if a list item applies to your research, read the appropriate section before selecting a response.

Materials & experimental systems

n/a	Involved in the study	
☐	☒	Antibodies
☐	☒	Eukaryotic cell lines
☒	☐	Palaeontology and archaeology
☒	☐	Animals and other organisms
☒	☐	Clinical data
☒	☐	Dual use research of concern
☒	☐	Plants

Methods

n/a	Involved in the study	
☒	☐	ChIP-seq
☒	☐	Flow cytometry
☒	☐	MRI-based neuroimaging

Antibodies

Antibodies used	Anti-RyR1 (F-1, Santa Cruz Biotechnology, used in 1:1000), Anti-calnexin(C4731, Sigma-Aldrich, used in 1:2000), HRP-conjugated anti-mouse IgG (04-18-18, KPL, used in 1:5000) and anti-rabbit IgG (074-1516, KPL, used in 1:5000)
Validation	Anti-RyR1 (F-1, Santa Cruz Biotechnology, used in 1:1000) was recommended for use in WB, IP, IF, and ELISA to detect ryanodine receptors from skeletal muscle, cardiac muscle, and brain tissues of mouse, rat, and human origins, as stated on the Santa Cruz Animal Health product page.<br>Anti-calnexin(C4731, Sigma-Aldrich, 1:5000) has been validated for use in immunohistochemistry and western blotting, as stated on the MilliporeSigma product page

Eukaryotic cell lines

Policy information about cell lines and Sex and Gender in Research

Cell line source(s)	Human Embryonic Kidney 293T cells (ATCC)
Authentication	None of the cell lines have been authenticated.
Mycoplasma contamination	Cell lines were not tested for mycoplasma contamination but no indication of contamination was observed
Commonly misidentified lines (See ICLAC register)	No commonly misidentified cell lines were used.

Plants

Seed stocks	No involvement of plant-related elements in our study.
Novel plant genotypes	No involvement of plant-related elements in our study.
Authentication	No involvement of plant-related elements in our study.
