# Supplementary material for: Cryo-EM structures of ryanodine receptors and diamide insecticides reveal the mechanisms of selectivity and resistance
Source: Nat Commun. 2024 Oct 20;15:9056. doi: 10.1038/s41467-024-53490-0 (PMC11491487; doi:10.1038/s41467-024-53490-0)
Supplement: Supplementary file 3 — Description of Additional Supplementary Files [file 41467_2024_53490_MOESM3_ESM.pdf]

Supplementary Movie 1.
Diamide-induced channel opening of chiRyR.

Supplementary Movie 2.
Conformational changes induced by the resistance mutations.
